# William Stern: The Relevance of His Program of ‘Differential Psychology’ for Contemporary Intelligence Measurement and Research

**DOI:** 10.3390/jintelligence11030041

**Published:** 2023-02-21

**Authors:** Kristof Kovacs, Csaba Pléh

**Affiliations:** 1 Institute of Psychology, ELTE Eotvos Lorand University Budapest, 1053 Budapest, Hungary; 2 Department of Cognitive Science, Central European University, 1051 Budapest, Hungary

**Keywords:** Stern, IQ, differential psychology, ipsative testing

## Abstract

William Stern is mostly renowned for inventing the IQ formula. However, he is also the originator of the term ‘differential psychology’ itself. His program of differential psychology synthesized population-based correlational studies as well as idiosyncratic approaches focusing on unique profiles of individuals. We argue that his approach still offers valuable ideas to this day; in particular, the individualistic sub-programme of Stern’s differential psychology corresponds to a large extent to ipsative testing that emphasizes a profile-based analysis of individual strengths and weaknesses.

## 1. Introduction

For most psychologists, even in the field of human intelligence, William Stern is solely renowned for inventing the IQ formula. However, his contribution to the study of human intelligence is much more widespread. For instance, Stern is the originator of not only the concept of IQ, but also of the term differential psychology itself (Jarl 1958). Moreover, later in his career, he eventually became a sharp critic of IQ and IQ testing, the very concept he is most remembered for.

In this paper, we present an overview of Stern’s intelligence-related work, including a discussion of his less well-known contributions. However, our aim is not to provide a purely historical account; on the contrary, we purport to highlight the surprising relevance of Stern’s ideas for contemporary research and application. Furthermore, it is not our intention to thoroughly introduce early differential research and its pioneers, such as Galton, Cattell, Terman, or Yerkes; our sole focus is on Stern and his legacy.

## 2. William Stern, a German Polymath

Louis William Stern (Berlin, 29 April 1871—Durham, North Carolina, USA, 27 March 1938) was an influential German psychologist and philosopher. A professor at Hamburg University between 1916 and 1933, his work was crucial in establishing the psychology of personality as a discipline. Stern came from a very educated rabbinate German Jewish family and received a thorough education in both the humanities and the natural sciences. He was a student of Hermann Ebbinghaus, an objective experimentalist, and Moritz Lazarus, an early proponent of idealistic *Völkerpsychologie*. All throughout his career, Stern was under the influence of both of these approaches: that of objective, empirical psychology and that of the tradition focusing on structures of meaning (Lamiell 1996, 2003).

Stern was a multi-faceted author, with a rare combination of theoretical and applied interests. For instance, he was a leading theoretician of early developmental psychology, emphasizing the active role of the child in the development of the mind (Kreppner 1992). Early on in his career he was involved in the progressive education movement, campaigning, for instance, for the coeducation of male and female students, but he also contributed to establishing the first laboratories in work psychology in Germany and the first institutions devoted to vocational psychology. He was also the first to study the reliability of eyewitness testimony (Stern 1903, 1910). Together with his wife, he was a pioneering researcher of child language development (Stern and Stern 1907); his framework for development focused on contextualization and the active role of the child in the process of language development. This monography was indeed a joint effort of the couple, reflected by the first authorship of the mother, who in fact created the diaries that served as the basis of the study. William Stern also published a pioneering case study of Helen Keller, the first deaf blind person who received higher education (Stern 1905). With his general system of personalism emphasizing the value of personality, he can also be interpreted as a forerunner of the positive psychology movement (Pléh 2004, 2006).

He fled Germany to avoid Nazi prosecution and accepted a professorship at Duke University in 1934. Unfortunately, due to his limited English as well as only four years spent in the United States before his death, he could not establish a differential psychology program there. As a result, his legacy was mostly discontinued.

## 3. Differential Psychology

Stern’s longest-lasting contributions were made in the field of individual differences. He declared the program of differential psychology in 1900 (Stern 1900) in the book *Über Psychologie der individuellen Differenzen (Ideen zu einer “differentiellen Psychologie”) (On the Psychology of Individual Differences: Toward a “Differential Psychology”)*. In a theoretical sense, his psychology of personality integrated the two inspirations from his student years: the nomothetic approach from Ebbinghaus and the individual and meaning-based idiographic approach represented among others by Lazarus and dividing German psychology at the time, especially in Berlin (see the contribution to this debate by his mentor, Ebbinghaus 1896). In this first version of *differential psychology,* Stern identified its three aims: to investigate the dimensions of inter-individual differences, to identify the causes of such differences, and to explore how the consequences of differences manifest themselves in schools or the workplace. This was an exhaustive description that covered most of the field of intelligence as it is known today. In Stern’s own words:

The tasks of differential psychology “form a trio, and involve the individual differences themselves, their causes, and their modes of expression. So the first question is: What are the differences? In what manner do individuals, peoples, etc. distinguish themselves from one another? […] The second question may be formulated: What causes the differences? Here, inquiry will be focused on the relationship of psychological characteristics to objective factors such as inheritance, climate, social position, rearing, adjustment, etc […] Thirdly, one can ask: How are the differences manifested? […] In its most general terms, the result of inquiry in this direction would be a psychological symptomatology and diagnostic.” (Stern 1900, pp. 4–5; translation Lamiell’s, from Lamiell 2003, p. 35).

In this first synthesis of differential psychology, Stern distinguished between two approaches. The first, *variation research,* focuses on a single trait and studies “formal regularities that are entailed in the very fact of mental variation” (Stern 1911, p. 8). The second, *correlational research,* explores the correlations between traits. Interestingly enough, the same duality was emphasized by Binet, too, apparently independently of Stern:

“One can differentiate two great questions [in individual psychology]:

  1. To study how mental processes vary according to individuals, which are the variable properties of these processes, and what is the extent of these variations.

  2. To study the relationships between different mental processes in the same individual; are there mental processes that are more important than the others, to what extent can the different processes be independent of each other, and to what extent can they have a mutual influence among them?”.

(Binet and Henri 1896, p. 412)

Importantly, Stern’s approach was conceived as part of experimental psychology, not as the independent sub-discipline that it later evolved into: “As Stern envisioned it, this subdiscipline would not replace or in anyway compete with the general-experimental psychology formally established by Wilhelm Wundt (1832–1920) at Leipzig some two decades earlier, but would instead complement the general-experimental psychology by investigating (…) differences between individuals.” (Lamiell 2010, pp. 135–36).

**Figure 1:**
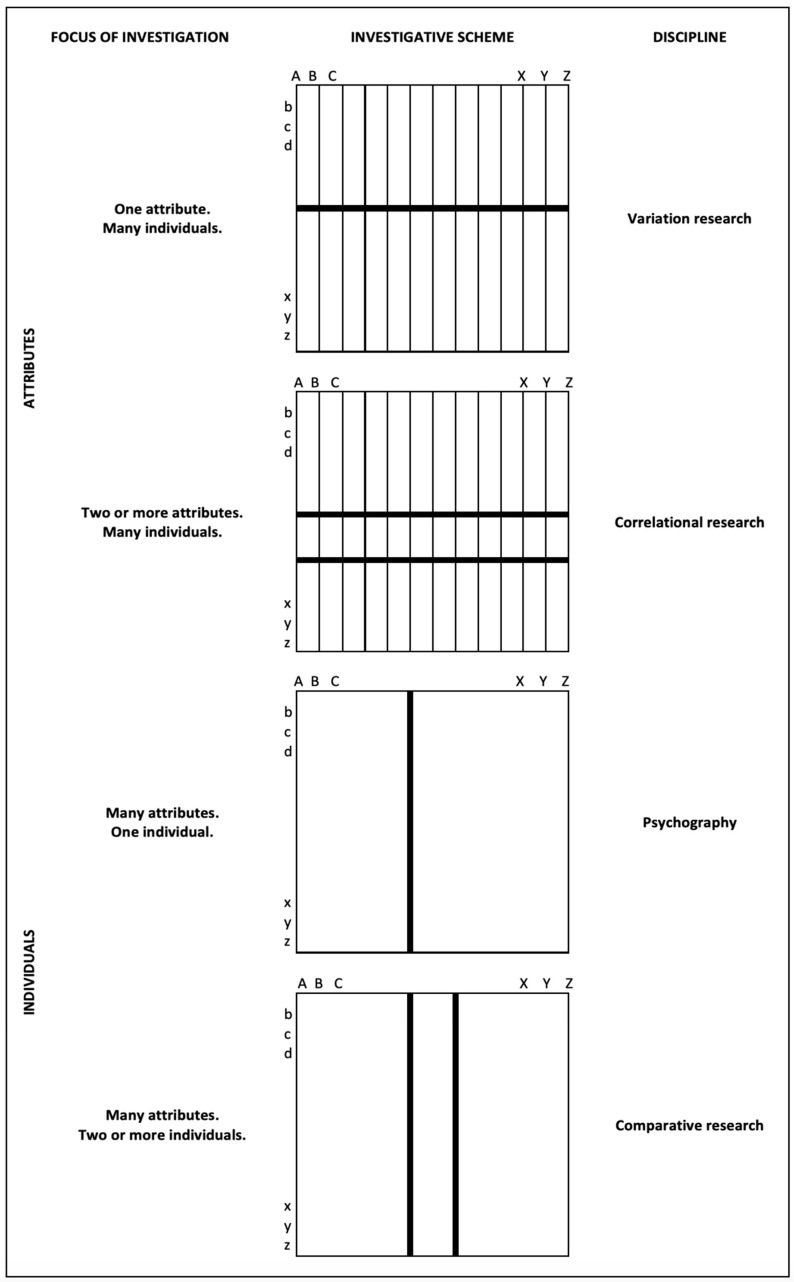
The four research schemes of differential psychology: variation research, co-variation research, psychography, and comparative research (after Stern 1911, p. 18).

In 1911, Stern published his second book on differential psychology: *Die Differentielle Psychologie in ihren methodischen Grundlagen* (*Methodological Foundations of Differential Psychology*). This was not simply a new edition, but an extension of his program. The 1911 version of differential psychology is illustrated in Figure 1. In Stern’s theorizing, differential psychology was the collection of four methods with two different orientations for research. As we have seen, the first two—variation research and correlational research—had already been established in the first version of the program, and both focused on *attributes* through the study of many individuals. Variation research focused on individual differences in a single trait, whereas correlational research focused on the covariance patterns between at least two traits. The latter conceptually covers the factorial studies of the structure of abilities and other kinds of multivariate analysis. In its 1900 version, only these two methods, variation and correlation research, constituted the approach.

Dissatisfied with the lack of focus on individuality in psychology, in 1911 Stern added two other methods that focused on *individuals* rather than attributes: *psychography*, the idiographic study of a single individual focusing on personal profiles, and *comparative research*, the comparison of two or more persons. These two additional methods corresponded to the idiographic attitude of the German humanist tradition. This school of personality research was introduced to American psychology by Gordon Allport (Allport 1937), with limited success.

## 4. Stern’s Influence

Unfortunately, only a handful of Stern’s works have been published in English (a notable exception is Stern 1914). As a result, Stern is mostly remembered in the history of psychology as “the IQ Guy” (Lamiell 1996). A telling piece of evidence of Stern’s underappreciation is that a paper surveying the history of differential psychology only introduces Stern on the sixth page and does not point out that he is in fact the originator of the term. Instead, he is mentioned as “another researcher whose work has not been as appreciated by Americans as much as it should” (Revelle et al. 2011, p. 9).

There are, of course, exceptions. In a paper discussing correlating persons versus correlating tests, Cyril Burt attributes this distinction originally to Stern: “These two complementary lines of approach have been termed by Stern the study of inter-individual and intra-individual variability, leading to what he terms ‘horizontal correlation’ and ‘vertical correlation’ respectively” (Burt 1937, p. 60). Burt’s paper, in turn, is referenced by Cattell’s seminal paper summarizing and comparing different approaches to factor analysis (Cattell 1952): Cattell, citing Burt’s summary of Stern, also attributes to Stern the core idea leading to the differentiation of person-oriented (P, Q) and trait-oriented (R) techniques.

**Figure 2:**
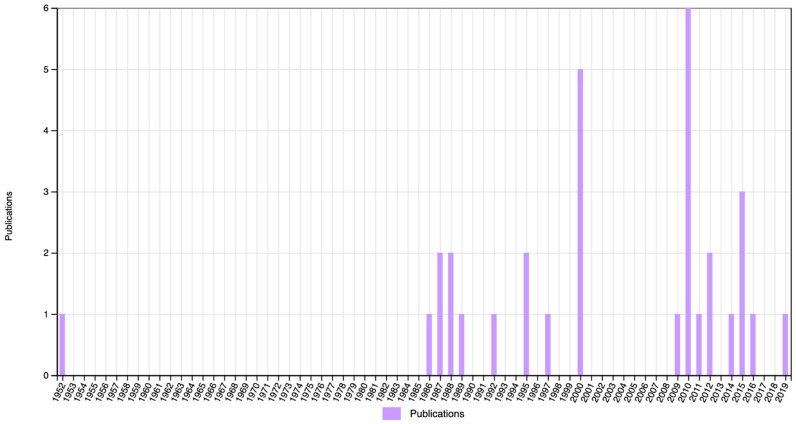
Number of publications mentioning ‘William Stern’ in the title, abstract, or keywords.

**Figure 3:**
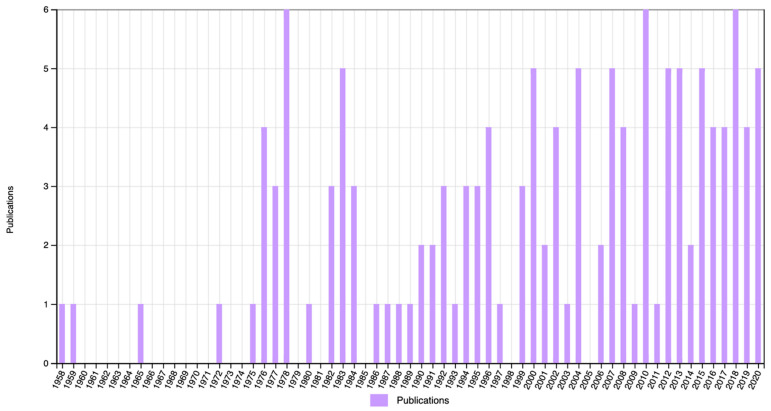
Number of publications mentioning ‘differential psychology’ in the title, abstract, or keywords.

**Figure 4:**
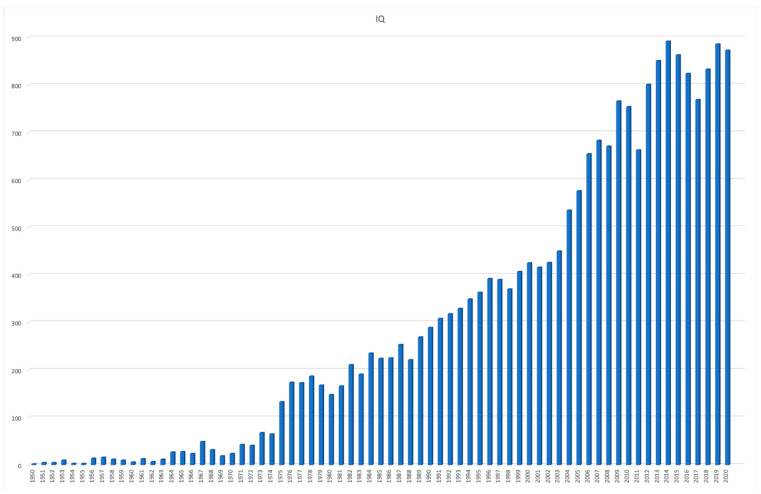
Number of publications mentioning ‘IQ’ in the title, abstract, or keywords.

However, it seems that Stern himself is less remembered by posterity than two of his main legacies: differential psychology and, in particular, IQ. On Web of Science, we performed a search on ‘William Stern’, ‘differential psychology’, and ‘IQ’ in titles, abstract, and keywords, in all publications in Psychology from 1950 to 2020 (Figure 2, Figure 3 and Figure 4). Stern was mentioned only once between 1950 and 1985, and even after that he is mentioned only sporadically. Differential psychology is mentioned with approximately the same frequency, at least since 1975, but still in no more than half a dozen papers in any given year. IQ, on the other hand, shows a constant increase all throughout the last 70 years.

## 5. Stern’s Approach to Intelligence Testing and Its Contemporary Relevance

Stern’s concept of the intelligence quotient (IQ) modified Binet’s calculation of the difference between mental age (Intelligenzalter) and chronological age to their ratio. The resulting quotient proved to be a more adequate indicator of development relative to peers than the original difference score, as IQ is “independent of the absolute magnitude of chronological age. The formula is, then: mental quotient ~ mental age ÷ chronological age. With children who are just at their normal level, the value is 1, with those who are advanced, the value is greater than unity, with those mentally retarded, a proper fraction” (Stern 1914, p. 42).

The concept of IQ became one of psychology’s most well-known ideas, and IQ tests are probably one of the most widespread psychological instruments. The IQ tests most widely used in educational and clinical practice are batteries that consist of numerous subtests with diverse content, and global ability indices are weighted sum scores calculated from the subtests or from lower-level indices. Examples of such global scores are FSIQ (Full-Scale IQ) in the Wechsler scales or the GIA (General Intellectual Ability) index in the Woodcock–Johnson Tests of Cognitive Abilities.

There are two different approaches to interpreting such global indices. A ‘top-down’ approach builds on the concept of ‘general intelligence’, which, in turn, is based on the positive manifold: the finding that scores on all cognitive tests correlate positively, and these all-positive correlations can be statistically accounted for by a general factor, *g*. In the ‘top-down’ approach, *g* represents general intelligence, a domain-general cognitive ability that, despite the superficially different content, is measured by all different subtests. IQ is a proxy for *g*; therefore, IQ, or any global index obtained from a test battery, is the most important indicator of cognitive performance. A ‘bottom-up’ approach puts larger emphasis on the actual tests and specific ability factors. Under this framework, global scores are conceptually identical to their technical manifestation: they are weighted averages, representing the average of the level of different cognitive abilities.

There is an additional but related dichotomy: the one between normative vs. ipsative assessment: relative strengths and weaknesses compared to others (normative) or compared to oneself (ipsative). It is possible that one’s relative strength is below the average of a norm group, but information on intra-individual strengths might still be useful for instruction and development. Kaufman’s idiographic *intelligent testing* approach emphasizes an ipsative, profile-based analysis (Kaufman 1994; Kaufman and Lichtenberger 2006) instead of only norm group comparisons on a global score. Arguably, profile-based assessment is much better suited for treatment or intervention purposes than a global, normative indicator. This approach concurs with Stern’s ideas regarding testing intelligence, which discouraged averaging results across different tests: “But just here does the value of Spearman’s method for the testing of intelligence become dubious. If we select four or five tests that show very high intercorrelations in order to use their totals as a measure of intelligence, there exists the danger that we may be testing by them only a very restricted portion of the field of intelligence and leaving entirely out of consideration other compensatorily important portions” (Stern 1914, pp. 113–14). Relatedly, he opposed relying on a single instrument when determining a person’s IQ: “no single test, no matter how good it may be, should ever be made the instrument for testing the intelligence of an individual” (Stern 1914, p. 19).

Paradoxically, while Stern invented the very concept of IQ, later in his career he became very critical of the exclusivity of normative testing (Lamiell 1996). This included a critique of his own IQ concept: “Seventeen years ago, when I introduced the concept of the ‘intelligence quotient’ as a measurement principle for intelligence tests, I had no idea that the ‘IQ’ would become a kind of worldwide formula and one of the most frequently encountered expressions in American jargon. (...) But beyond that, many additional tests (…) have now been developed, standardized, and put into use, (...) always with emphasis on the objective, quantitative norm, with reference to which the single case is then compared.” (Lamiell 2012, p. 380). One of the most important things to learn from his later work is his focus on individual mental profiles that challenge the dominance of global, standardized indicators in mental testing.

An ipsative approach breaks exactly this exclusivity of normative testing; hence, it is quite possible that Stern would have approved of it. Of course, psychography—his approach to focusing on individuality—was supposedly a non-statistical approach, but methods for ipsative testing were naturally not available at the time. It is not unreasonable to assume that Stern would have endorsed this method as a legitimate technique under psychography—especially since he emphasized the importance of idiosyncratic profiles and pointed out that two persons with the same IQ usually have very different underlying profiles. Additionally, he had an interest in psychological methods for career advising, which is one of the areas where ipsative assessment greatly compliments normative results (Stern 1938).

## 6. Conclusions

Kaufman’s classic book, *Intelligent testing with the WISC-R,* which popularized the ipsative approach, does not have Stern in its author index (Kaufman 1979). Under the intelligent testing approach, the ‘‘global IQ on any test, no matter how comprehensive, does not equal a person’s total capacity for intellectual accomplishment’’ (Kaufman and Lichtenberger 2006, p. 20). Amazingly, this is almost identical to Stern’s treatment from 1938: “‘an intelligence quotient’ may be of provisional value as a first crude approximation when the mental level of an individual is sought; but whoever imagines that in determining this quantity he has summed up ‘the intelligence’ of an individual once and for all, so that he may dispense with the more intensive qualitative study, leaves off where psychology should begin’’ (Stern 1938, p. 60).

In this paper, we hope to have demonstrated the importance and relevance of Stern’s contributions to contemporary research and how his ideas seem to be relevant for substantive debates in the field of differential psychology as well as for practical issues in cognitive assessment and test score interpretation. We look forward to an increasing appreciation of Stern in psychology and in the field of intelligence in particular.
